# A Deep-Learning Approach to Detect and Classify Heavy-Duty Trucks in Satellite Images

**DOI:** 10.1109/tits.2024.3431452

**Published:** 2024-08-29

**Authors:** Xingwei Liu, Yiqiao Li, Langting Sizemore, Xiaohui Xie, Jun Wu

**Affiliations:** School of Information and Computer Science, University of California at Irvine, Irvine, CA 92697 USA; Department of Civil Engineering, The City College of New York, New York, NY 10031 USA; Program in Public Health, University of California at Irvine, Irvine, CA 92697 USA. She is now with California’s Smoking Cessation Program, Orange County, Santa Ana, CA 92701 USA; School of Information and Computer Science, University of California at Irvine, Irvine, CA 92697 USA; Department of Environmental and Occupational Health, Joe C. Wen School of Population and Public Health, Susan and Henry Samueli College of Health Sciences, University of California at Irvine, Irvine, CA 92697 USA

**Keywords:** Port-side heavy-duty trucks, deep learning, object detection, satellite images, GIS, model ensemble

## Abstract

Heavy-duty trucks serve as the backbone of the supply chain and have a tremendous effect on the economy. However, they severely impact the environment and public health. This study presents a novel truck detection framework by combining satellite imagery with Geographic Information System (GIS)-based OpenStreetMap data to capture the distribution of heavy-duty trucks and shipping containers in both on-road and off-road locations with extensive spatial coverage. The framework involves modifying the CenterNet detection algorithm to detect randomly oriented trucks in satellite images and enhancing the model through ensembling with Mask RCNN, a segmentation-based algorithm. GIS information refines and improves the model’s prediction results. Applied to part of Southern California, including the Port of Los Angeles and Long Beach, the framework helps assess the environmental impact of heavy-duty trucks in port-adjacent communities and understand truck density patterns along major freight corridors. This research has implications for policy, practice, and future research.

## I. Introduction

Heavy-duty trucks are one of the essential elements of the roadway network. They play a vital role in the economy and supply chain [1], [2] and have significant impacts on energy consumption [3], greenhouse gas, and pollutant emissions [4]. In order to understand the impacts of heavy-duty trucks on many aspects of society, researchers and practitioners are increasingly interested in obtaining heavy-duty trucking data with a higher spatial resolution, especially in areas like container ports, and major freight gateways.

Many data sources have been explored in the literature to understand heavy-duty truck movements, including survey data, mobile sensor data, and stationary sensor data. The U.S. Census Bureau collected the Vehicle Inventory and Use Survey (VIUS) data from 150,000 trucks (including lightweight and heavy-duty trucks) which are registered to individuals and small businesses to provide data regarding the physical and operational characteristics of the national truck population [5]. The data collection of the latest survey lasted for around eight months. However, VIUS is collected every five years [5]. It is hard to capture the dynamic spatial distribution of truck activities, especially at the neighborhood level. On the other hand, mobile sensor data such as the Global Position System (GPS) is capable of capturing the spatiotemporal distribution of heavy-duty trucks and has been adopted to estimate statewide truck origin-destination information [6], to analyze truck travel behavior [7], and to obtain truck performance measures at critical freeway corridors [8]. However, the GPS probe vehicles may provide biased truck samples, since the truck GPS data used by most studies are collected mainly from larger fleet companies [6] who provide limited details due to privacy concerns. Alternatively, many transportation agencies utilize stationary sensors, and other axle detection technologies such as piezo sensors [9] to obtain truck classification counts along major freight corridors. However, the spatial distribution is limited because of their high installation and maintenance costs. Conversely, inductive loop sensors are widely deployed across the United States highway network and have a longer service life. Inductive loop sensor systems have the ability to provide truck classification [10], [11] when these existing loop sensor systems are equipped and updated with inductive signature technology and advanced machine learning models. However, such technology has limited coverage on local arterial roads, off-road container yards, warehouses, and other freight facilities that are considered hot spots for understanding trucking demands, air pollution, and potential emission impacts generated by heavy trucks.

Traditional traffic data collection methods focus on obtaining traffic volume at freeway segments [9], [10], [11], [12]. It is difficult for such methods to capture vehicle distributions in larger regions. With the advancement of satellite imagery technologies, researchers started to use satellite images on various transportation applications [13]. At the early stage of satellite image-based vehicle detection studies, researchers utilized handcraft features from satellite images to identify vehicle objects [14], [15]. With the prosperous development of deep learning models in recent years, convolution neural networks (CNN) have been gradually introduced to the task of vehicle detection on satellite images. Researchers used a graph-based superpixel segmentation to detect the position of the vehicles and then trained a CNN to distinguish vehicle and non-vehicle objects [16]. A study in Egypt explored the use of Faster Region CNN (Faster R-CNN) and Single Shot Multi-Box (SSD) to detect vehicle objects on a set of satellite images collected from multiple sources such as the World-View from Maxar and Google Earth which purchases images from multiple operators including Maxar [17]. The image resolution of the overall dataset in their study is less than 1 meter [17]. The Faster R-CNN presents a better average precision value of 0.89 for vehicles (mixture of trucks and passenger vehicles). Stuparu et al. proposed a one-stage object detection by adopting RetinaNet architecture for detection vehicles using satellite images [18]. They developed a vehicle detection model on the Cars Overhead with Context (COWO) dataset with a resolution of 0.15 meters and had a mean average precision (mAP) of 0.72 [18]. To further improve the precision of the vehicle detection model, Ozturk and Cavus used the same dataset as [18] and designed a lightweight hybrid vehicle detection method [19]. They constructed a CNN model to predict vehicle objects on the satellite images and then refined the detection results through the use of threshold filters and morphological operations. This hybrid approach achieved a precision value of 0.96. However, from the transportation application perspective, two major gaps exist in the literature. Firstly, existing open pre-labeled datasets such as COWO [20], Common Objects in Context (COCO) [21], and Remote Sensing Super-resolution (RSSOD) [22], have relatively high resolution but are limited to specific small study areas and time of day, while commercially available datasets that cover larger spatial regions usually have lower resolutions. Thus, the models developed on a high-resolution open dataset are expected to have a significant degradation in performance when they are adopted to detect vehicles on a newly collected low-resolution dataset. Second, the existing detection frameworks mainly targeted general vehicle objects and lack an understanding of various heavy-duty trucks and their distributions at local communities, truck corridors, container terminals, etc, which are essential locations of interest to analyze the environmental and public health impacts of heavy-duty trucks [23].

To fill this gap, we developed a heavy-duty truck detection framework through the fusion of satellite images and publicly-available GIS information to better detect and classify port-side heavy-duty trucks and shipping containers and further estimate their density distribution across major truck corridors, port terminals, local warehouses, and residential communities. First, a modified CenterNet was designed to detect and classify trucks and shipping containers that can be classified into 5 categories. According to the location of the heavy-duty trucks and containers, objects are categorized into on-road and off-road heavy-duty trucks/shipping containers. The off-road heavy-duty trucks are further classified based on two general sizes for their containers (20ft and 40–53ft) and the status of the containers (being part of container trucks and standalone containers). An additional class called “Other parked heavy-duty trucks” was designed for the parked trucks which are neither container trucks nor shipping containers. Then, an ensemble of the CenterNet [24] and Mask R-CNN [25] was designed to improve the model performance. Finally, a GIS-based detection filter was applied to refine the detection results from the ensemble model. This proposed detection framework presents promising results with an average precision value of 0.89 for limited-resolution satellite images at 0.5 meters resolution.

The rest of the paper is organized as follows. Section II introduces the satellite images used in this study and describes the definition of every labeled object. Section III details the overall design of the truck detection framework through the fusion of a satellite image-based object detection algorithm and a GIS location filter. Section V demonstrated a showcase of using the truck detection framework to estimate the on- and off-road truck distribution at the Long Beach port area. In the last section, we conclude this study with discussions and future works.

## II. Data Description

**Fig. 1.**
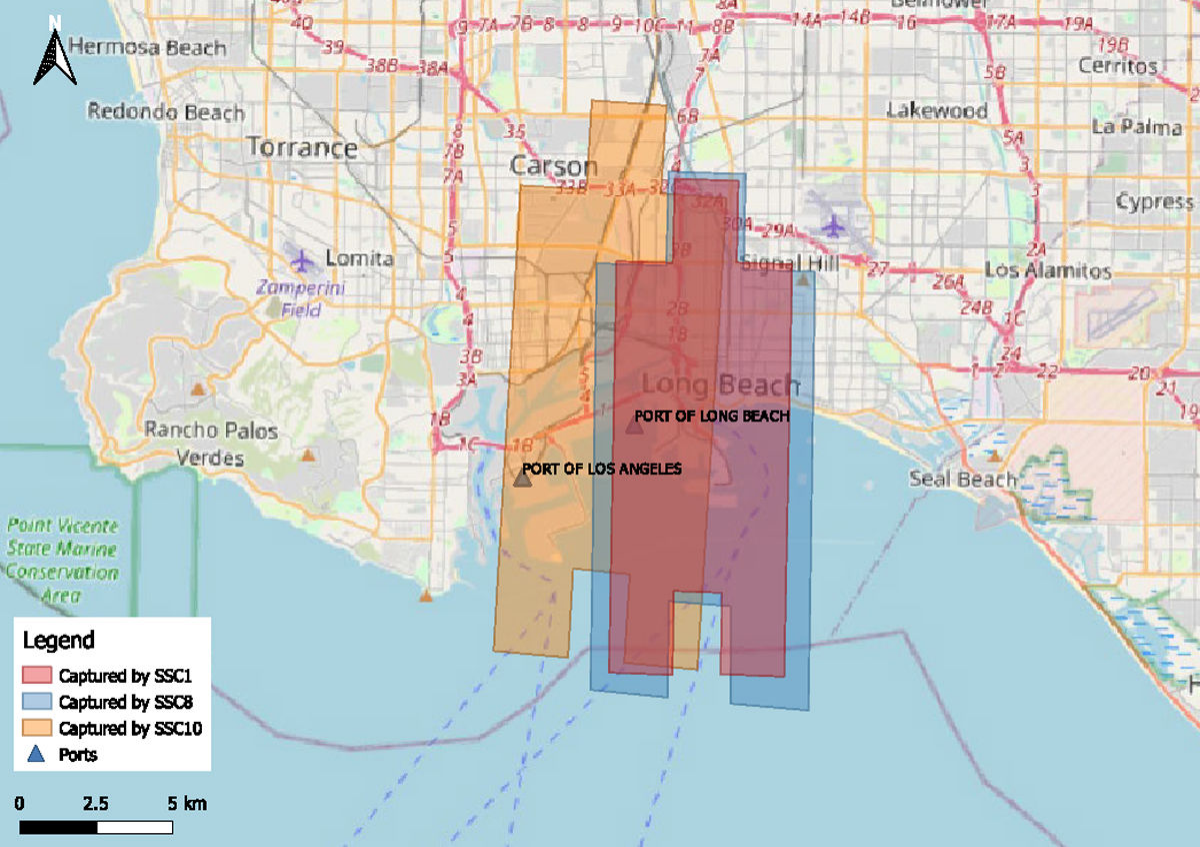
Map of the coverage areas of 3 satellite images. Coverage areas of images 1 to 3 are rendered in red, orange, and blue respectively.

The satellite images utilized in this study, with a ground sample distance (GSD) of 0.5 meters, were captured by the SkySat constellation, consisting of 21 high-resolution Earth imaging satellites owned and operated by Planet Labs PBC [26]. In this dataset, multiple satellite sensors take the bird’s-eye-view of the study area at different timestamps which allows the possibility of capturing the dynamic patterns of truck distribution at different times of day. Three satellite images were acquired and prepared for the model development, and the size of each image is presented in Table I. Every satellite image was cut into 512 × 512-pixel tiles. Truck objects in each tile were labeled by oriented bounding boxes through manual visual verification. Table I presents the summary of each satellite image that was used in this study. Figure 1 shows the visualization of the coverage areas.

In this study, we aim to understand the distribution of on-road and parked heavy-duty trucks and shipping containers near the Port of Long Beach and Los Angeles, where many container trucks are operated, and shipping containers are parked. We designed our classification scheme with a focus on the container trucks and shipping containers as well as their locations. We categorized heavy-duty truck objects into five major classes including on-road/parked container trucks and standalone container boxes with two different size groups, and one “other heavy-duty trucks (P)” to capture other off-road heavy-truck types that are not container trucks. The detailed class description and their training and testing sample size are presented in Table II, where (*P*) and (*R*) represent the truck objects in the parking facilities and on the road respectively. All data is manually labeled and objects are represented by 4-vertex polygons. Due to the difficulties in identifying objects in limited satellite images, we added cross-checking and a second round of labeling to make sure the labeled objects are consistent. While training, we processed the data so that the closest rotated rectangle is used as the training ground truth for each object.

**Fig. 2.**
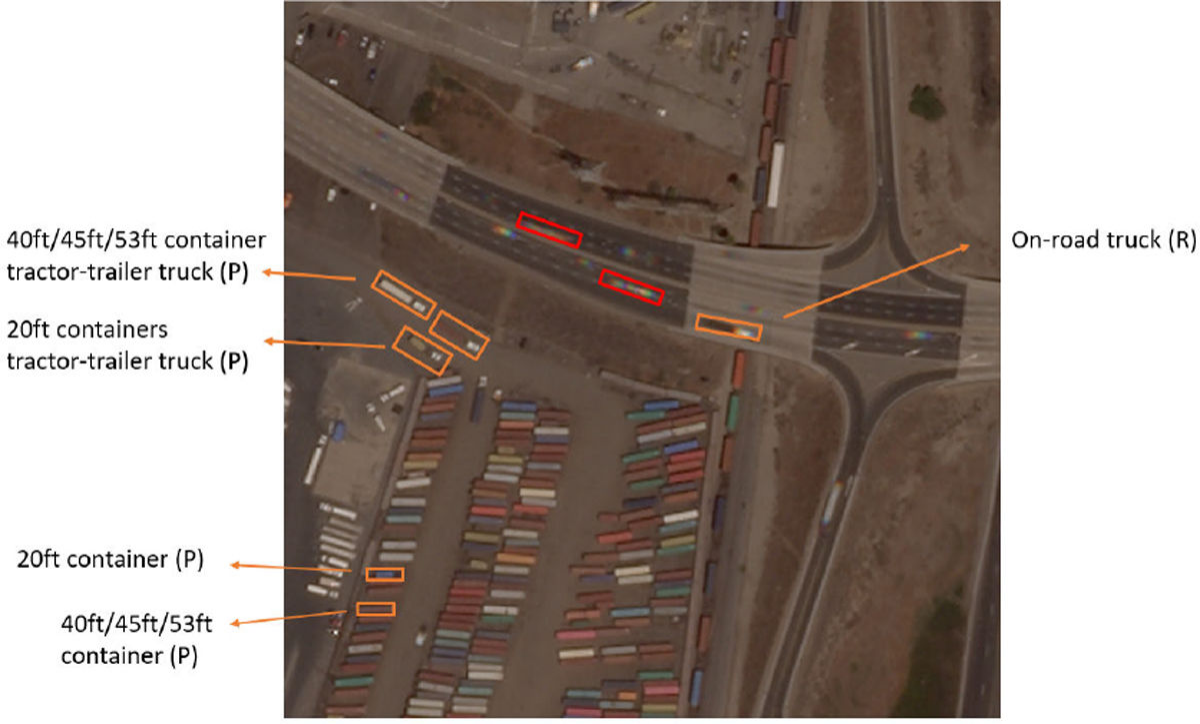
Truck object illustration.

Figure 2 demonstrated the visual representation of the heavy-duty truck and container objects. As shown in the red boxes in Figure 2, the color displacement that is shown at the tail of the fast-moving trucks on the road makes the truck objects challenging to be distinguished from container trucks to the other body configurations. Therefore, we aggregated and labeled all on-road heavy-duty trucks as one single category. In addition, it is hard to visually distinguish 40ft and 53ft containers. Thus, these two sizes of containers were combined in this study.

## III. Methodology

**Fig. 3.**
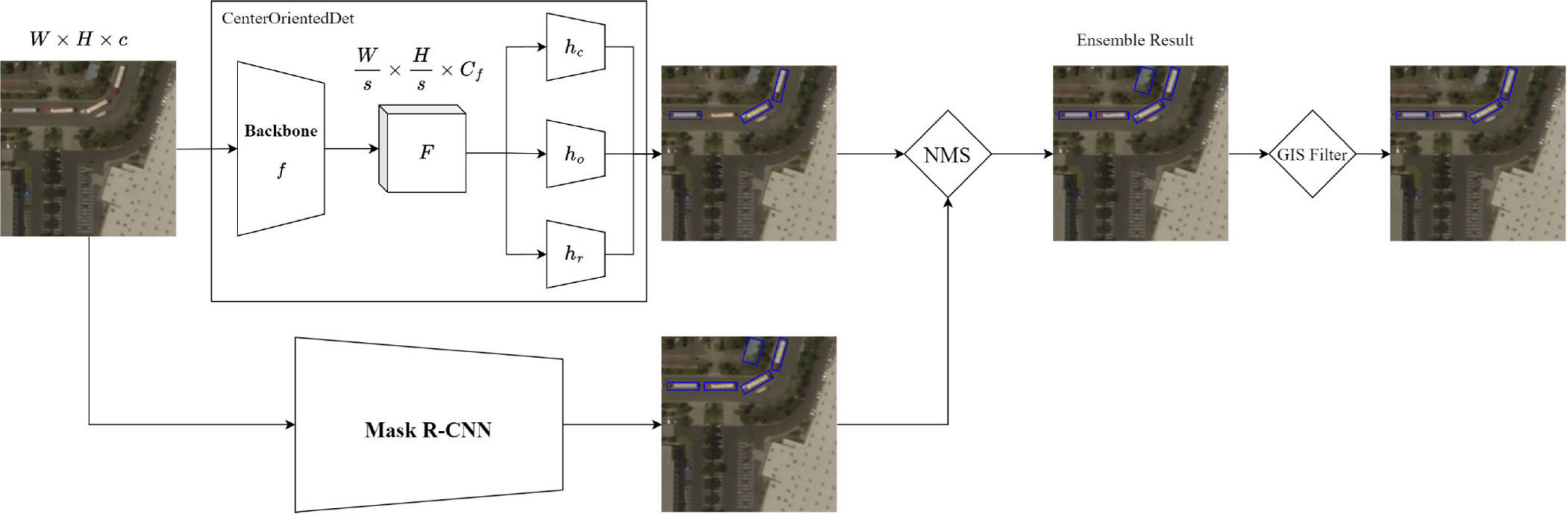
The overall pipeline of the proposed framework. Note that the detection bounding boxes are simulated to illustrate the functionality of our pipeline. $W,\;H,\;C,\;f,\;s,\;C_{f},\;h_{c},\;h_{o},\;h_{r}$ are meta-parameters of the proposed model, as defined in Section III-A1. NMS is Non-Maximum Suppression, which will be introduced in Section III-A4.

This study designed a novel truck detection framework comprising a Truck Object Position Detector (TOPD) and a GIS-based Detection Filter (GIS-DF). The structure of the proposed framework is presented in Figure 3. First, the TOPD utilized an ensemble of a center-corner-based object detection and an instance segmentation model to identify the location and class information of each truck object in the satellite images. Then a mapping algorithm calculates the geographical position of each detected truck from its image position. Lastly, the OpenStreetMap [27] API used to query geographical information of the detected truck objects and to filter out false detections located in unreasonable areas, such as in the ocean or on-road trucks that are far away from roads.

### A. Truck Objects Position Detector (TOPD)

The main component of the TOPD is an object detection model, which takes in image tiles and predicts the locations and sizes of desired objects in the images. With the recent advancement of neural network architectures, deep learning methods are widely used in object detection tasks given their higher effectiveness compared to the traditional image processing methods [28]. There are two major types of deep learning-based object detection models: two-stage detectors and one-stage detectors. A typical two-stage detector like the R-CNN [29] family consists of a region proposal extractor and a region classifier. The region proposal extractor first extracts all the predefined regions,^1^ which are called anchor boxes, covering the whole image at various possible combinations of positions and sizes, then further classifies if each region contains the desired object in a certain category. Later work like Faster-RCNN [30] replaces the region proposal algorithm with a neural network to first roughly identify possible anchor boxes according to their image content to reduce the work of the classification stage.

With the later development of deep learning models, inferencing object information directly from an image without an extra step of region proposal became more widely used because of a simpler pipeline and higher inference speed [31], [32]. Those single-stage detection frameworks use different methods to formulate the detection problem but generally consist of a heavy feature extractor (backbone), an optional aggregation connection between backbone outputs (neck), and a series of simple convolutional networks to execute the desired task (head).

As indicated in Table VIII, recent advancements in single-stage detectors have significantly narrowed the gap in accuracy between the two approaches while maintaining a higher inference speed. This makes them more suited for real-time applications.

CenterNet [24], a recent single-stage framework, employs an anchor-free approach. This model directly regresses bounding box sizes and classes at each pixel location, generating heatmaps from the input image. This design simplifies the network architecture, making it more flexible and easier to implement compared to models with custom detection modules like SSD and YOLO. While it is important to recognize some limitations of the anchor-free method, such as potential precision trade-offs, for our specific application in detecting trucks in image tiles, the benefits of speed, accuracy, and simplicity offered by CenterNet make it the most appropriate choice. Therefore, in this study, we adopt CenterNet [24] as the foundation of the TOPD, considering its balance between inference speed, detection accuracy, and simplified network architecture.

#### 1) Center-Corner Based Detection Structure:

Formally, the detector takes in a colored input image $I\in P^{H\times W\times C}$, where P is the pixel value range, H is the image height, W is the image width, and C is the number of the color band for the image. In our dataset, we set $P\;=\;\left[0,\;1\right]$, W=512, H=512,C=3. Without losing generality, the backbone is defined as a function $f\;:P^{H\times W\times C}\to R^{H_{f},W_{f},C_{f}}$ that calculates a 3D tensor F=f(I), which is also called extracted features, from the input image. $H_{f},\;W_{f},\;C_{f}$ are the shape of F and $H_{f},\;W_{f}$ are usually proportional to the input image shape H,W, or $H=sH_{f},\;W=sW_{f},\;s\geq 1$. The exact value of s is determined by the number of strides of convolution and pooling operations in the feature extractor. In this study, we use ResNet 101 [33] as our feature extractor to extract critical features from the images and set s=8 in the model.

Subsequently, multiple heads are used for making predictions based on the extracted features from the backbone model. Each head, defined as $h\;:R^{H_{f}\times W_{f}\times C_{f}}\to R^{H_{f}\times W_{f}\times C_{h}}$, consist of a 3 × 3 convolutional layer with ReLU [34] activation, and a 1 × 1 convolutional layer. The final output channel $C_{h}$ is defined according to the usage of the output heads.

In the rest of this section, the formal definition of the network inference process will be described, given an input pixel coordinate (x,y) with corresponding output coordinate after the head $\left(x^{o},\;y^{o}\right)=(\lfloor x/s\rfloor ,\lfloor y/s\rfloor )$.

##### a) Class prediction head:

The first head is the class prediction head $h_{c}$. The output of the first head is the probability of a bounding box centering at a pixel location exists given the bounding box class and pixel location. The output of $h_{c}$ is shown as follows:

(1) $$h_{c}(F)\in R^{H_{f},W_{f},C_{c}}$$

Formally, given a pixel location (x,y) in the original input image, the probability of an object of class cls exists at that point is

(2) $$Pr\left(\exists bb_{cls}|x,\;y,\;I\right)=h_{c}(F{)}_{x^{o},y^{o},cls}$$

where $0\leq cls<C_{c}$ is the number representing a certain class.

##### b) Center offset head:

The pixel in the final output cannot be accurately mapped to the original image due to the scaling factor s. An offset is required to compensate for the error between the location of a predicted center and a real center: $x-sx^{o},\;y-sy^{o}$. The offset is predicted by a dedicated head $h_{o}$:

(3) $$R^{H_{f}\times W_{f}\times C_{f}}\to R^{H_{f}\times W_{f}\times 2}$$

Thus, if $\left(x^{o},\;y^{o}\right)$ is a center point according to Equation 2, the coordinate of the real center point $\left(x_{ct},\;y_{ct}\right)$ in the input image can be written as follows:

(4) $$x_{ct}=s(x^{o}+h_{o}(F{)}_{x^{o},y^{o},0})$$

(5) $$y_{ct}=s(y^{o}+h_{o}(F{)}_{x^{o},y^{o},1})$$

##### c) Corner regression head:

**Fig. 4.**
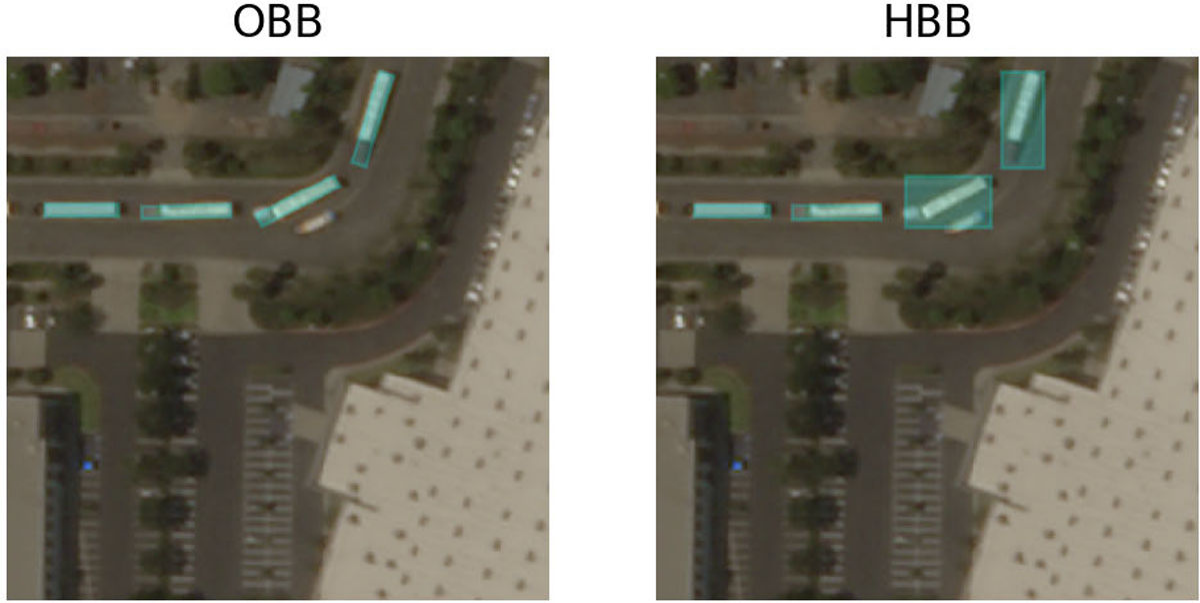
Differences between Oriented Bounding Box and Horizontal Bounding Box.

With the center location, the final bounding box could be regressed by an extra head $h_{r}$. In the original design of CenterNet [24], the bounding boxes are assumed to be parallel to the axes, but we found that this assumption does not apply to satellite images. In normal photographs, objects tend to follow the direction of gravity, which is usually parallel to the axes. However, in satellite images, the alignment of the objects has no such tendency, as shown in Figure 4. Thus, in our framework, we designed a head $h_{r}$ to predict the offset of each corner point relative to the center point instead of only predicting width and height values. The designed head $h_{r}$ is written as:

(6) $$R^{H_{f}\times W_{f}\times C_{f}}\to R^{H_{f}\times W_{f}\times 8}$$

Thus, the final bounding box prediction, which centers at output coordinate $\left(x^{o},y^{o}\right)$, is a polygon and defined by a set of vertices $V_{x^{o},y^{o}}=\left\{\left(x_{i},\;y_{i}\right)|i=0,\;1,\;2,\;3\right\}$. The $x_{i}$ and $y_{i}$ values are written as follows

(7) $$x_{i}=s(x^{o}+h_{o}(F{)}_{x^{o},y^{o},0}+h_{r}(F{)}_{x^{o},y^{o},2i})$$

(8) $$y_{i}=s(y^{o}+h_{o}(F{)}_{x^{o},y^{o},1}+h_{r}(F{)}_{x^{o},y^{o},2i+1})$$

#### 2) Loss Functions:

The loss function [24] is defined by three components: classification loss $L_{c}$, regression loss $L_{r}$, and offset loss $L_{0}$. The **classification loss** is a combination of Focal Loss [35] and logistic regression given total number of objects N in the training dataset, where ${\overset{ˆ}{Y}}_{xyc}=h_{c}(F{)}_{x^{o},y^{o},c}$:

(9) $$L_{c}=-\frac{1}{N}\sum_{x^{o},y^{o},c}\left\{\begin{array}{cc} {\left(1-{\hat{Y}}_{xyc}\right)}^{\alpha }\log({\hat{Y}}_{xyc}) & \text{if}\;Y_{x^{o},y^{o},c}=1\\ {\left(1-Y_{x^{o},y^{o},c}\right)}^{\beta }{\left({\hat{Y}}_{xyc}\right)}^{\alpha } & \text{otherwise}\\ \log(1-{\hat{Y}}_{xyc}) & \end{array}\right.$$

**Fig. 5.**
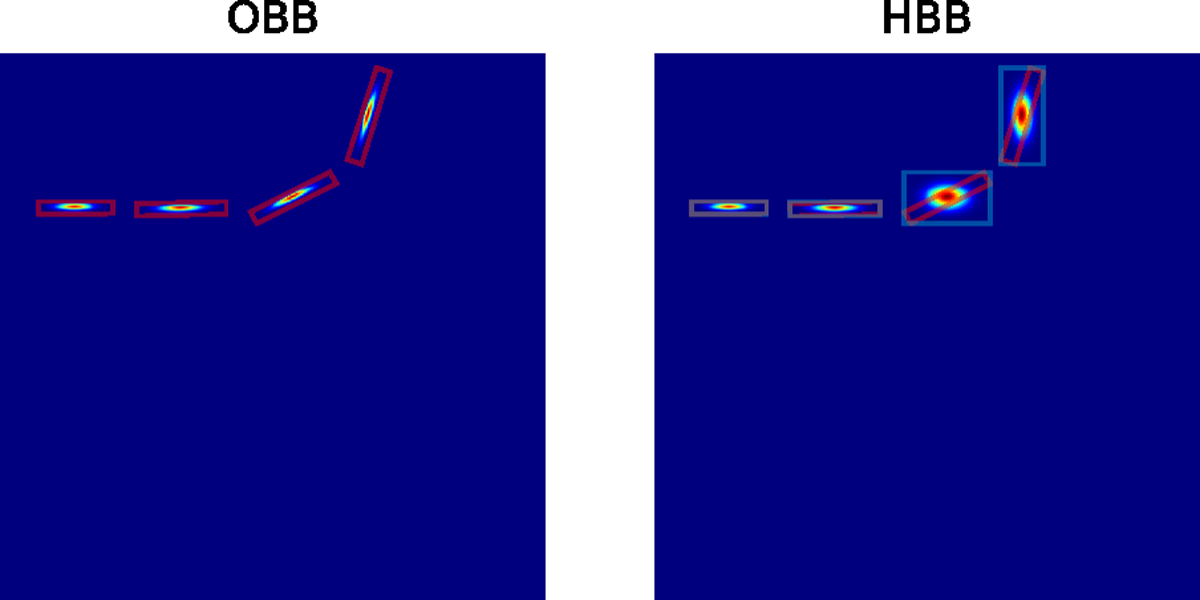
Visualization of center heatmaps of Oriented Bounding Box and Horizontal Bounding Box. Red box: oriented bounding boxes. Cyan box: horizontal bounding boxes. The image used here is the same image used in Fig. 4.

The classification loss defined in CenterNet [24] is calculated based on a Gaussian center confidence heatmap generated from ground truth bounding boxes. In our work, since all the bounding boxes could be rotated in the satellite image, we employed a rotated Gaussian center heatmap generation algorithm to let the detector focus on the object features. As visualized in Fig. 5, our center heatmap is a rotated version of the Gaussian heatmap generated by the procedure defined in CenterNet [24]. Formally, $Y_{x^{o},y^{o},c}$ is the ground truth probability map of class c, while pixel (x,y) is the center of an object of class c. The rotated Gaussian center heatmap is generated by a Gaussian function around the true center and rotated to align with the bounding box.

(10) $$Y_{x^{o},y^{o},c}=\underset{p}{max}\;exp\left(-\frac{{\left(x^{\prime }-\left\lfloor p_{x}/s\right\rfloor \right)}^{2}}{\sigma_{x}^{2}}-\frac{{\left(y^{\prime }-\left\lfloor p_{y}/s\right\rfloor \right)}^{2}}{\sigma_{y}^{2}}\right)$$

where p is the center of each bounding box, and $\left(x^{\prime },y^{\prime }\right)$ is the distance between the pixel $p^{\prime }=(x,y)$ and two adjacent edges of the bounding box in the pixel space of $h_{c}(F)$. More specifically, assume the bounding box consists 4 vertices $v_{0},\;v_{1},\;v_{2},\;v_{3}$ in clockwise order, $x^{\prime }=\frac{\left|\left(v_{0}-v_{3}\right)\times \left(v_{3}-p^{\prime }\right)\right|}{\|\left|v_{0}-v_{3}\right||},\;y^{\prime }=\frac{\left|\left(v_{2}-v_{3}\right)\times \left(v_{3}-p^{\prime }\right)\right|}{\left\|v_{2}-v_{3}\right\|}.\;\sigma_{x}=\frac{\left\|v_{2}-v_{3}\right\|}{5},\;\sigma_{y}=\frac{\left\|v_{0}-v_{3}\right\|}{5}$ are to determine the level of diffusion of the Gaussian heatmap.

The **offset loss** $L_{o}$ is a L1 loss:

$$L_{o}=\frac{1}{N}\sum_{p}\left|h_{o}(F{)}_{\left\lfloor p_{x}/s\right\rfloor ,\left\lfloor p_{y}/s\right\rfloor }-\left(\frac{p}{s}-\left[\left\lfloor p_{x}/s\right\rfloor ,\left\lfloor p_{y}/s\right\rfloor \right]\right)\right|$$

The **regression loss** $L_{r}$ is similar to $L_{o}$, extending L1 loss to 4 corner points:

$$L_{r}=\frac{1}{N}\sum_{k=1}^{N}\sum_{j=1}^{4}\left|BB_{k,j}-v_{j}\left(BBc_{k,x},BBc_{k,y}\right)\right|,$$

where BB is the set of all true bounding boxes, and BBc is the center point of each element in BB.

Finally, the full loss is defined by

(11) $$L=L_{c}+\lambda_{r}L_{r}+\lambda_{o}L_{o}$$

where $\lambda_{r}$ and $\lambda_{o}$ are parameters controlling the scale of each loss.

#### 3) Training Details:

We used the mmdetection project [36] to implement several key modifications as discussed in the previous section.

1. Adapted Heatmap Generation: We have employed rotated Gaussian heatmaps to accommodate the varying orientations of trucks.
2. Customized Loss Functions: The classification and regression losses have been modified to align with the rotated heatmaps and the direct regression of bounding box corners, ensuring more accurate truck detection. The dataset was preprocessed before training.

Specifically, images were split into 512 × 512 pixel tiles as detailed in the Data Description (Section II). During the training process, we employed several data augmentation techniques to improve model robustness and handling of limited and unbalanced data:

- Random flip with a 50% chance on both horizontal and vertical axes.
- Random rotation by multiples of 90 degrees.
- Random adjustments in brightness, contrast, and saturation within a range of 50% to 150%.

We trained the model for 60,000 iterations with 64 images per batch. For training parameters, we followed [24] and set $\lambda_{r}=0.1,\;\lambda_{o}=1$. The overall learning rate was set to 0.01 with a cosine annealing scheduler on a Stochastic Gradient Descent optimizer [37].

#### 4) Model Ensemble:

As shown in columns labeled as Det from Table III, the CenterNet-based detection model performs well on most parked trucks but less effective on on-road trucks, which is the only class representing moving trucks and is an important class for conducting truck-related research. As shown in Section II, on-road trucks have blurry boundaries and are usually thinner due to the “tail” effect, which is difficult for a one-stage detection model to identify. Thus, we explored a 2-stage segmentation model (Mask R-CNN [25]) for its potential to better handle difficult cases. The Mask R-CNN [25] is trained directly on the data by converting oriented bounding boxes to segmentation polygons. The results in Table III show a significantly better performance for on-road trucks over the segmentation model. As the performance of the segmentation model is worse than that of the detection model for the other classes, we used an ensemble method to combine the result together from the two models. The model ensemble is a classical method to improve model performance and generality in classification tasks [38], [39]. The usage of an ensemble in multi-model object detection is still new but effective [40]. We followed [40] and used non-maximum suppression to combine the result from different models.

**Fig. 6.**
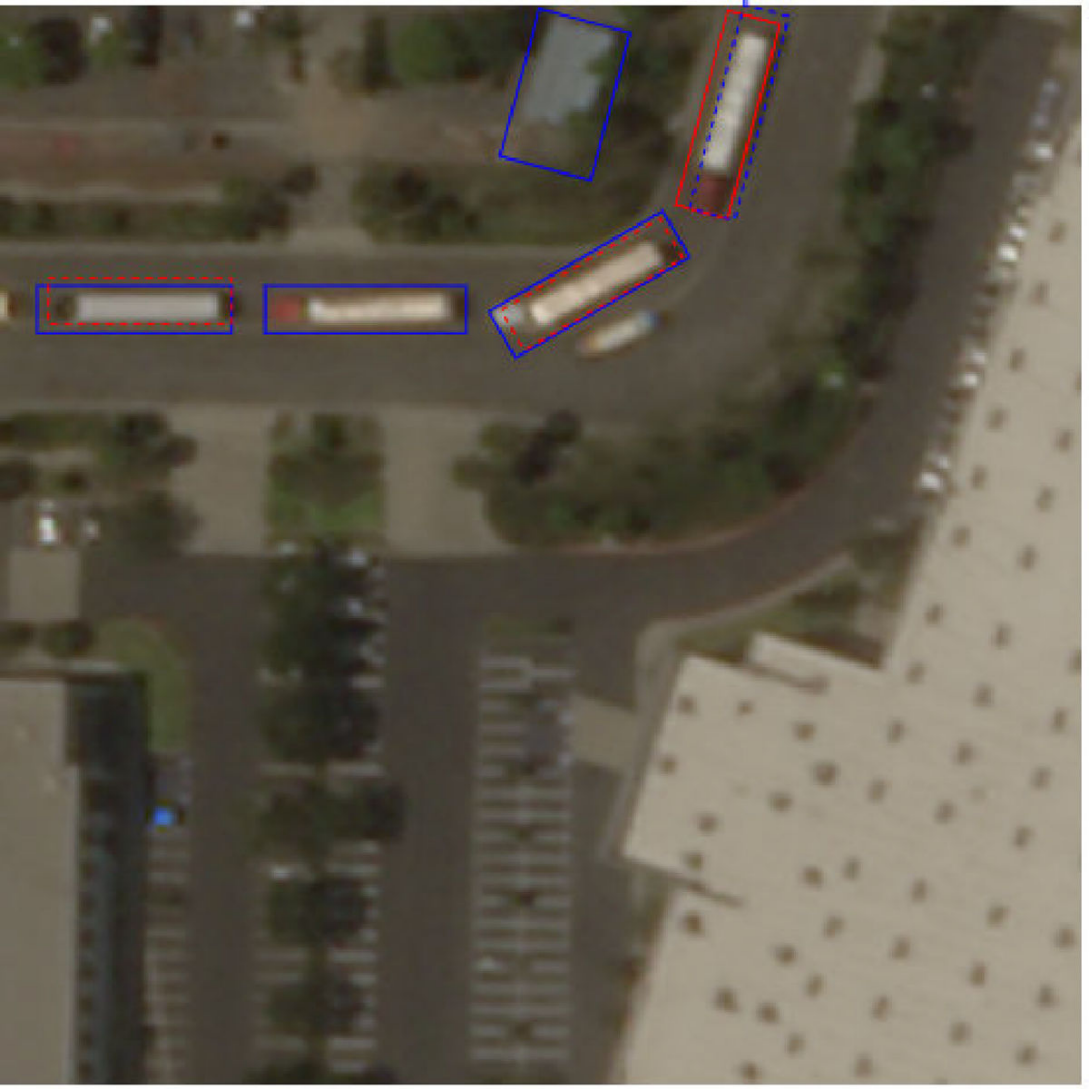
Visualization of the Non-Maximum Suppression process. The colors of the boxes represent which model predicts the proposal bounding boxes. Red: detection model. Blue: segmentation model. Dashed boxes are the ones getting filtered out because there is another box with higher prediction confidence nearby (solid boxes).

As shown in Figure 6, the predicted bounding boxes from two models are merged and filtered through the process of non-maximum suppression based on the center offset defined in Section III-C for each class. Specifically, given all of the predicted bounding boxes $bb_{i}=\left(c_{i},\;k_{i},\;p_{i},\;V_{i}\right)$ from both models where $c_{i}$ is the predicted class, $k_{i}$ is the confidence level from the model, $p_{i}$ is the center, and $V_{i}$ is the set of vertices of the predicted bounding box. The final set of predictions BB is defined as:

(12) $$BB=\left\{bb_{i}|\left\{bb_{j}|j\neq i,c_{j}=c_{i},\left\|p_{i}-p_{j}\right\|<2,k_{j}>k_{i}\right\}=\emptyset \right\}$$

### B. GIS-Based Detector Filter GIS-DF

Accurately identifying the geographic location of heavy-duty trucks and distinguishing on- and off-road trucks is an essential task for our detection frameworks, as they play dissimilar roles in transportation applications and yield different environmental and public health impacts on local communities. For example, the on-road truck data can be used to identify truck bottlenecks to provide strategic guidelines on transportation infrastructure upgrade [41], whereas the density of parked heavy-duty trucks and containers revealed the trucking demand at the study region. Further, off-road trucks may be located closer to residential communities and thus have higher noise and air pollution impacts on the community [42]. To further improve the performance of the on-road truck detection from the ensembled detection model, we integrated GIS information from OpenStreetMap (OSM) [27] into our framework. This step is applied as an optional post-processing procedure independent of the ensemble model. The data obtained from OSM [27] are a set of nodes 𝒩, a set of line segments $𝒮\in {𝒫}_{\mathrm{ordered}}(𝒩)$, and a set of relations ℛ∈𝒫(𝒮) defined the road network consisting of segments, where 𝒫 means power-set. Each segment in 𝒮 represents the geometric shape of a road segment, and each element in ℛ represents all the segments belonging to the same road. Two connected segments in a relation share the same end node. From that information, given a coordinate +p, we can query the nearest segment s∈𝒮 to p with distance d. The distance is then used to further refine the detection results. For detections of *On-road truck* (R) class, the geometric center of the detected bounding box is calculated as p and if the distance d between p and the closest road center is larger than a predefined threshold $d_{0}=10$ meters, the detection is treated as a parked truck. A reclassification model is then used to classify the detection between *20 ft box truck (P)*, *40–53ft box truck (P)*, and *Other truck (P)*. On the other hand, for detected parked trucks, if the distance to the nearest road segment is smaller than $d_{0}$, it will be then assigned to *On-road truck (R)* class.

### C. Evaluation Metrics

The performance of the detection methods is generally evaluated by average precision (AP) and mean average precision (mAP) [43]. AP and mAP use intersection over union (IoU) between predicted bounding boxes and true bounding boxes to determine prediction correctness. However, in our dataset, truck objects are thin and less than 10 pixels wide, which means IoU-based evaluation is very sensitive to small prediction errors, even of 1–2 pixels. In addition, our focus in this work is the distribution and density of trucks in space, rather than the exact footprint a truck occupies. Thus, the IoU-based evaluation is not well-fitted for the application of understanding truck distribution in a large region. There, we modified the average precision measure by using a bounding box center offset instead of IoU. Formally, a true positive happens when $\|p-\overset{ˆ}{p}\|<\frac{\left\|{\overset{ˆ}{bb}}_{0}-{\overset{ˆ}{bb}}_{3}\right\|}{10}$, where p is the predicted center point, $\overset{ˆ}{p},\;\overset{ˆ}{bb}$ is the true center point and bounding box vertices.

## IV. Model Results

We evaluated the performance of our framework as well as the impact of each component on a randomly selected 100 cropped tiles at testing data from three satellite images listed in Table I. All testing tiles are not included in the training process. Each cropped tile has a dimension of 512×512 pixels. The tiles are selected with biased probabilities to ensure each class has a minimum of 30 objects in the test set. The final distribution of testing and training data is listed in Table II. In this section, we will discuss the framework performance and impact of each framework component in detail.

### A. Results Analysis

**Fig. 7.**
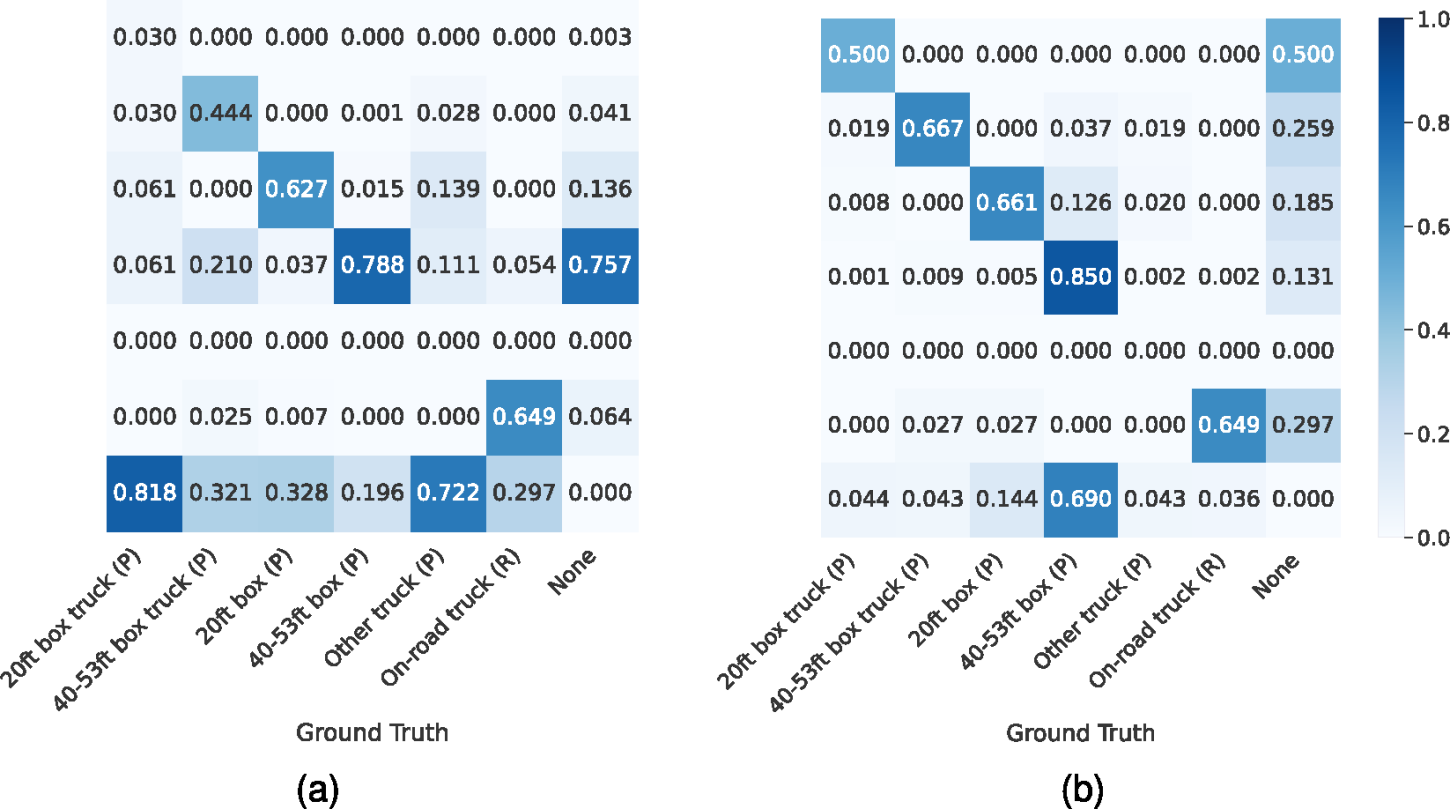
Confusion matrices displaying the model’s performance at a confidence threshold of 0.6, excluding the raw number of predictions. Sub-figure (b) is normalized by the number of actual objects in each ground truth class, while sub-figure (c) is normalized by the number of predicted objects for each class. These matrices provide insight into the model’s precision and recall, facilitating a detailed analysis of the classification accuracy for different truck categories.

As shown in Table III, the average precision numbers fluctuated across different classes. This is the result of unbalanced data distribution in the training data. According to Table II, the number of objects in *40–53 box (P)* class represents 82.9% of the training samples while minor classes like *Other truck (P)* and *20ft box truck (P)* only represent 0.1% and 0.3% of training samples. This heavily unbalanced data results in better fitting in *40–53 box (P) class* and poor fitting on the aforementioned two minor classes. The segmentation model performs better in detecting on-road trucks but does not match the performance of the detection model in other classes. The ensemble model has the best overall performance as well as the best performance among 4 out of 6 classes. From the results shown in Table III, the GIS-based filter improves the on-road truck detection performance across all three different variations of models. On every variation of our detector, the average precision improves by a large margin of more than 4, with a maximum of 7.13. The performance damage on other classes is less than 0.6 AP. This result shows that the introduction of a GIS-based filter is effective against on-road truck detection while maintaining performance on other classes. This post-processing step is used as a standard procedure for our framework in later discussions about framework use cases. We also conducted error analysis on each class by calculating the confusion matrix, shown in Figure 7. From Figure 7(b), the false positive rate is more than 0.25 for *20ft box truck (P)*, *40–53ft box truck (P)*, and *On-road truck (R)*. Also the false negative rate is high for *20 ft box truck (P)* and *Other truck (P)* as shown in Figure 7(a). Those 4 classes are the ones with the fewest total number of objects as shown in Table II. This shows that the model performance could be improved by introducing more data on those minor classes.

### B. Model Robustness Analysis

In our domain shift analysis, we diverged from the standard practice of using a random subset of tiles. Instead, we employed the entirety of Image 1 as the validation set. This image, captured in a similar area but at a different time compared to Images 2 and 3, offered a unique opportunity to assess the model’s adaptability to temporal variations within the same geographical region. Due to data constraints, as we only had access to these three images, our analysis could not extend to spatial domain shifts (i.e., different geographical areas). Our findings, as summarized in Table IV, reveal a nuanced picture of the model’s performance under these conditions. Notably, the model maintained usable accuracy levels for the *40–53ft box (P)* class, the most represented category in our dataset. This suggests that the abundance of training data in this class contributes to its resilience against temporal shifts. Conversely, the performance drop in the *On-road truck (R)* class was relatively modest. This might be attributed to the distinctive visual features associated with moving trucks, such as road texture and motion blur, which aid in their identification despite temporal changes in the imagery. This smaller decline in performance under domain shift conditions indicates that increasing the training data, especially for less represented classes, could enhance the model’s overall robustness. By providing more varied examples, the model could better learn to distinguish similar objects across different times, potentially improving accuracy in scenarios with limited representation. These results, while constrained by the available data, offer valuable insights into the model’s capabilities and limitations. They underscore the need for a more diverse dataset, which could further enhance the model’s adaptability to both temporal and spatial domain shifts. In addition to our domain shift analysis, we conducted a further robustness check by resampling a new training and validation split from our dataset. This experiment was designed to test the consistency of our model’s performance across different data partitions. The newly sampled validation set was approximately twice the size of the original validation set, providing a more robust evaluation of the model’s capabilities. The results of this experiment, as integrated into Table IV, confirm the model’s consistent performance across both the original and the resampled validation sets. Notably, the model’s accuracy levels in the resampled validation test, particularly for the *40–53ft box (P)* and *On-road truck (R)* classes, aligned closely with those observed in the original validation set. This consistency in performance across different data splits indicates that the model’s effectiveness is not a consequence of the specific data partitioning but a reflection of its inherent robustness and reliability. These findings enhance our confidence in the model’s robustness and its ability to generalize across different data samples. They also highlight the model’s potential effectiveness in broader applications, reaffirming the importance of diversified training data for achieving reliable performance in varying scenarios.

### C. Comparison With Contemporary Detection Models

To contextualize our framework’s performance, we compared it with recent state-of-the-art models: Oriented RCNN [44], Rotated RepPoints [45], and Oriented RepPoints [46], focusing on truck detection in satellite imagery. As Table V shows, each model was trained from scratch using our dataset for a fair comparison.

Our model exhibited superior performance, especially in detecting specific truck classes, outperforming other models in overall combined AP scores. This comparative analysis not only demonstrates our model’s efficacy but also highlights its unique strengths in satellite-based truck detection, suggesting avenues for further research and development.

Our results indicate that while the model performs well in detecting prevalent classes, it shows limitations in detecting minor classes such as ‘On-road truck (P)’. This could be attributed to the unbalanced nature of the training data. To address this, future work could focus on incorporating a more diverse set of images, encompassing a wider range of environmental conditions and object distributions.

## V. Case Studies

Traditional infrastructure-based truck data collection methods such as weigh-in-motions [12], piezoelectric sensors [9], and inductive loop detections [10], [11] are only able to capture truck data along major highways at a specific point, which miss heavy-duty trucks and shipping containers parked at the residential communities and near local warehouses. Those off-road trucks and shipping containers could imply the trucking demands and potential adverse environmental and health impacts on the local communities. In addition, it is hard for traditional point detectors to capture truck density on a continuous roadway network. This section demonstrated a case study that uses our framework for both on- and off-road heavy-duty truck detections. First, we applied our detection framework to the Port of Los Angeles and Long Beach area. Further, the truck density distribution was overlaid with the CalEnviroScreen 4.0 [47] data, an environmental justice screening tool developed by the California Office of Environmental Health Hazard Assessment of the California Environmental Protection Agency [47]. The CalEnviroScreen integrated 13 indicators representing pollution burden (e.g. fine particulate matter, diesel particulate matter, traffic impacts) and 8 indicators of population vulnerability (e.g. asthma, cardiovascular disease, poverty, unemployment) at the Census tract level for the State of California. The State designated the community with the overall CalEnviroScreen score ranking above 75% as disadvantaged communities. In this study, we compared the distribution of heavy-duty trucks and shipping containers in disadvantaged versus non-disadvantaged communities near the port area and discusses the environmental injustice issues. Second, we used our detection framework to estimate the heavy-duty truck distributions on both major freeways and surface streets and then zoom into a major freight corridor - I-710 to characterize the pattern of on-road heavy-duty trucks in local residential communities at different time of day.

### A. Community-Wise Truck Density Estimation

In this section, we present an example of using satellite images to analyze truck density in the Long Beach area and examine its correlation with pollution burden and population vulnerability metrics provided by CalEnviroScreen 4.0 [23]. CalEnviroScreen 4.0 describes potential exposures to pollutants and adverse environmental conditions caused by pollution, focusing on disadvantaged communities as identified by California Senate Bill 535 [48].

Building upon the methodology introduced in the previous section, we compare the distribution patterns of trucks and containers at different times by conducting our analysis on two distinct satellite images, image 1 and image 3 from Table II. We chose these images due to their large overlapping area to maximize the coverage of our analysis, which includes 32 out of 38 census tracts classified as disadvantaged communities [48].

Our focus area consists of the intersection of these two satellite images, and we filtered all detected trucks and census tracts with less than 80% of their area inside the survey area. We applied our framework to calculate the number of heavy-duty trucks and containers (Table II) per square mile. The truck density for each tract area was computed as the number of heavy-duty trucks or shipping containers per square mile. Following this analysis, we will discuss the implications of our findings and their relevance to understanding the relationship between truck density and pollutant concentration.

Table VI reveals a relatively small number of trucks compared to containers in the survey area. Furthermore, image 3 has a significantly lower number of objects overall. This discrepancy may be attributed to image 3 being captured on a Saturday, resulting in fewer truck activities. Among the 38 census tracts in image 3, 23 have no trucks detected, and containers are present in 10 of those tracts. In contrast, image 1 shows 7 tracts without trucks and 4 tracts with neither trucks nor containers. Considering the low truck volumes in image 3, total 71 trucks in 38 census tracts is too limited to get meaningful statistics from the data. Thus, we incorporated both trucks and containers in the remainder of this case study.

In addition to the previously mentioned differences in truck and container quantities, our analysis shows a very weak correlation (−0.05 < *R* < 0.05) between the number of trucks and containers in the survey areas. This low correlation suggests that the two variables do not directly influence each other. Although containers themselves do not produce pollutants, their presence can serve as an indirect indicator of truck activity in the area, as they are typically associated with cargo transportation. By including containers in our analysis, we can account for potential discrepancies in truck volume data and provide a broader context for understanding the relationship between truck activity and pollutant concentration. This approach ensures that our study captures a more comprehensive picture of the factors influencing pollutant levels, strengthening our ability to draw meaningful conclusions from the data.

We qualitatively demonstrated the relationship between the distribution of predicted heavy-duty truck and shipping container density and the CalEnvironScreen [23] pollution burden in Figure 9. Figure 9 (a), (b), and (c) display a similar color scale pattern, where census tract areas with higher heavy-duty truck and shipping container densities generally exhibit higher pollution burden scores. In image 1, 28.9% of the tracts have more than 100 trucks per square mile, 60.5% of the tracts have 1–99 trucks per square mile, and 10.5% of the tracts have no detected trucks. However, the maximum density reaches 2074.6 trucks per square mile, representing a long-tail distribution.

**Fig. 8.**
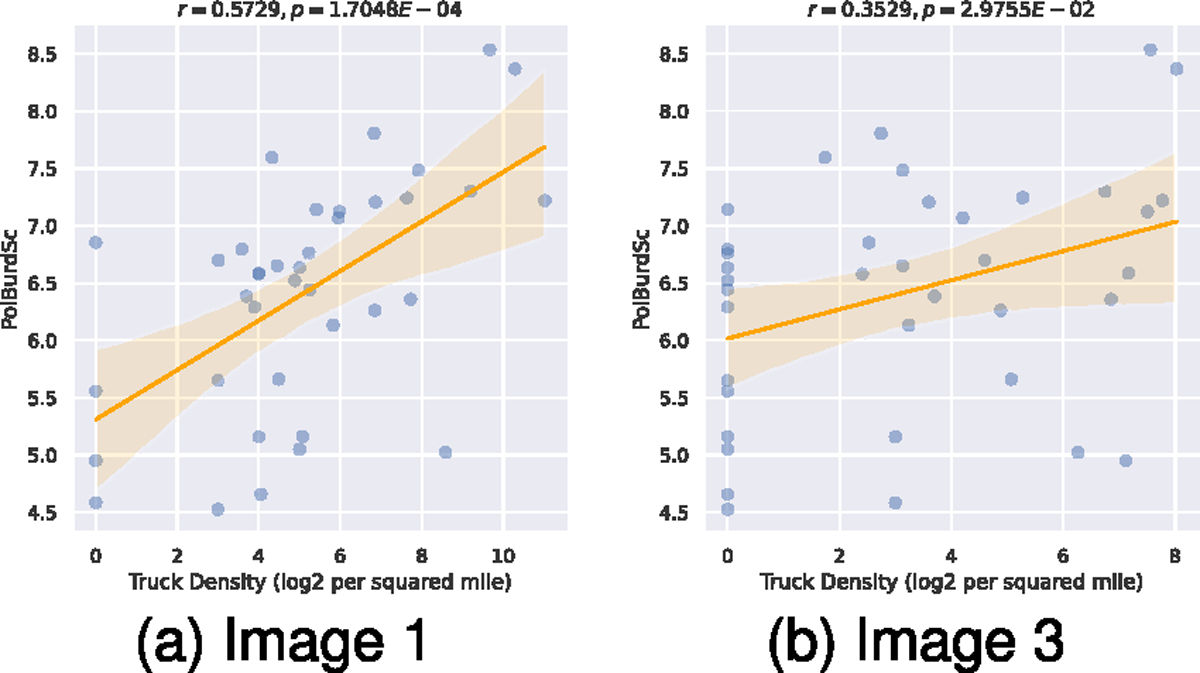
Correlation analysis over truck density against pollution burden. Yellow band represents 95% confidence band.

**Fig. 9.**
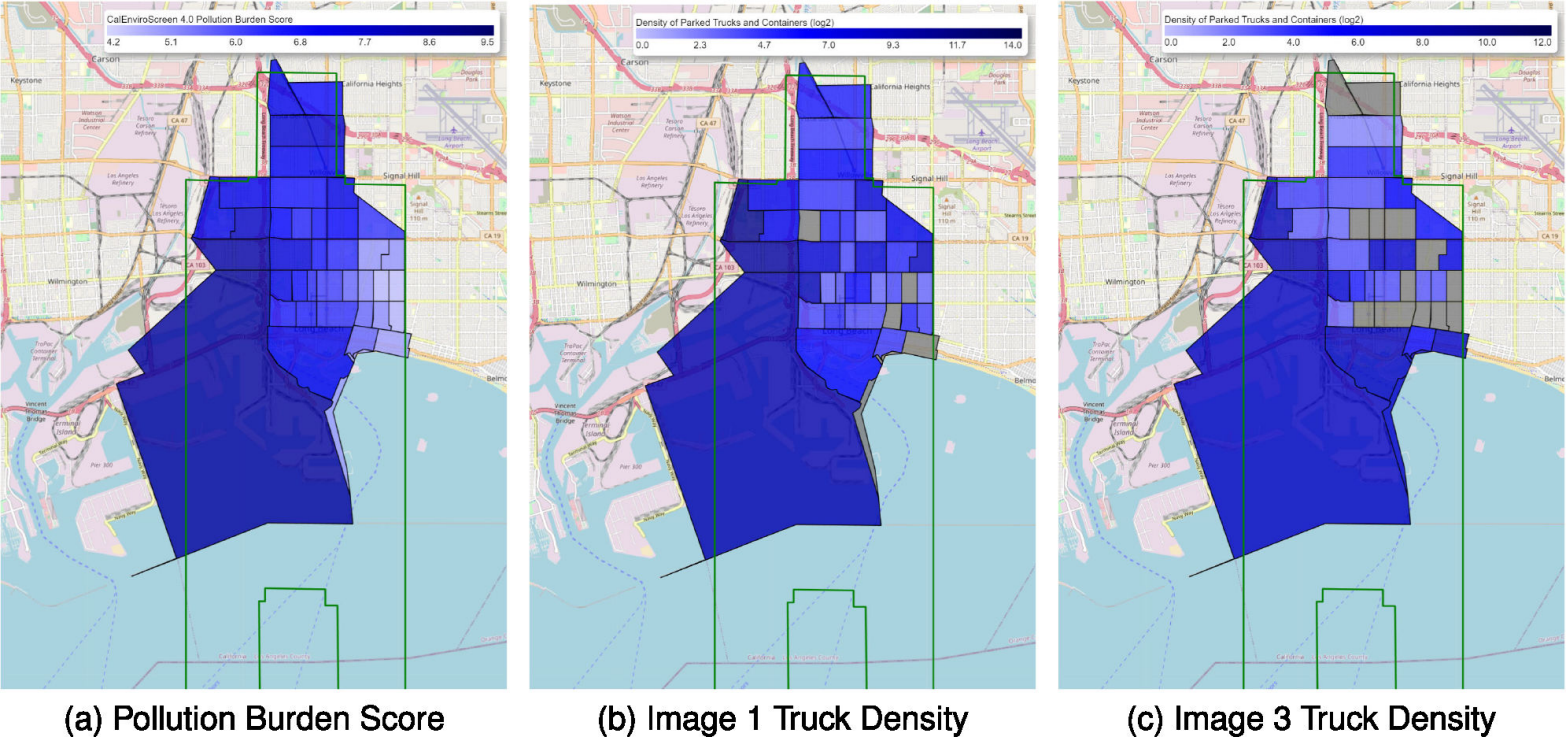
Comparison between off-road truck/container density (a) and pollution burden (b,c) in each census tract within the survey area. Gray areas have no truck detected. Green polygon is the survey area.

To better examine the correlations, we used *log*_2_ to transform the density values and discarded all tracts with zero truck detections. The results are shown in Figure 8. The *log*_2_ of truck and shipping container density correlates linearly with the pollution burden score, with a Pearson correlation coefficient of *R* = 0.573 and *p* = 1.7 × 10^−4^ for image 1 and *R* = 0.353*, p* = 2.98 × 10^−2^ for image 3. The narrow width of the confidence band indicates a good fit for the linear relationship.

We also conducted a quantitative analysis on image 1 using 23 different CalEnviroScreen 4.0 metrics, presenting the results with *R* > 0.2 in Table VII. The detailed correlation graphs can be found in Appendix B. The table reveals significant correlations between truck and container density and environmental metrics such as hazardous waste, underground water tank leaks, and pollutants in water bodies. These correlations suggest that areas with higher truck and container densities may experience increased exposure to pollutants and environmental hazards. Unemployment rate also exhibits a positive correlation with truck and container density, indicating a potential relationship between truck-related activities and socioeconomic conditions. The moderate correlation of the truck and container density with the CalEnviroScreen pollution burden score likely reflects the impact of historical land-use policies that caused many undesirable or environmentally hazardous facilitates being built in this area.

Furthermore, from a social equality perspective, we compared the distribution of heavy-duty trucks and containers across disadvantaged and non-disadvantaged communities (Census tracts receiving the highest 25 percent of overall scores in CalEnviroScreen 4.0 vs. those with scores below the top 25 percent). Our estimation results indicate that disadvantaged communities have an average density of heavy-duty trucks and containers approximately three times higher than non-disadvantaged areas. The density value is 334.9 heavy-duty trucks and containers per square mile in disadvantaged communities, compared to 88.47 in non-disadvantaged communities.

### B. On-Road Truck Traffic Analysis

Interstate 710 (I-710) is one of the busiest heavy-duty truck corridors in the U.S. which connects local freight facilities and the nation’s two largest ports, the Port of Los Angeles and the Port of Long Beach. It has also been considered a “Diesel death zone” because it carries more than 200,000 vehicles average daily volume, where approximately 34% of them are heavy-duty diesel container trucks according to the data from 2021 LA County Goods Movement Strategic Plan [49]. There are several residential communities located along I-710 and being exposed to the pollutant emission from the on-road container trucks. Therefore, this case study focuses on estimating the on-road heavy-duty density distribution along I-710 to provide data support on environmental-related policymaking.

**Fig. 10.**
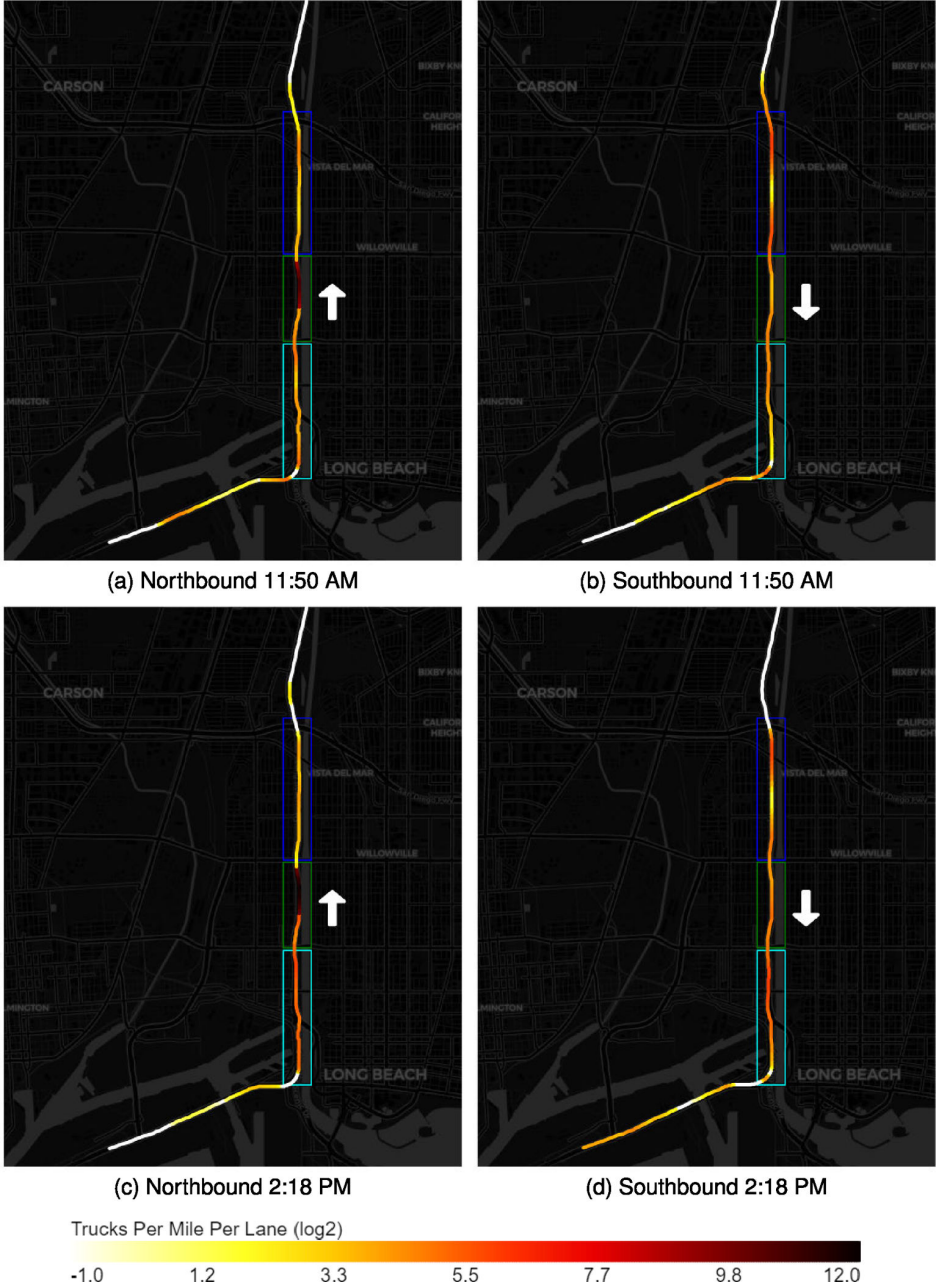
Truck distribution on I-710. The white arrow indicates the road direction.

**Fig. 11.**
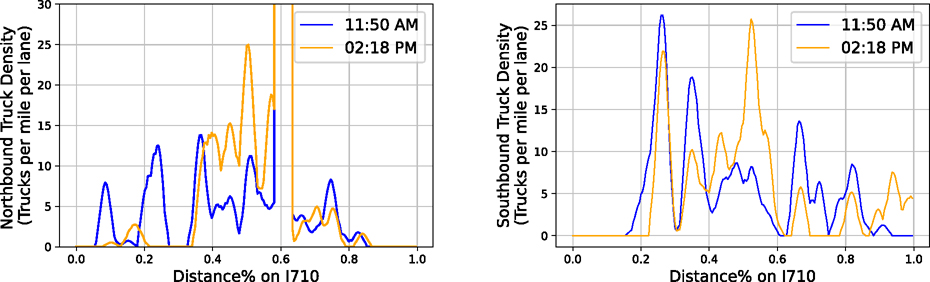
This graph compares truck densities at different times (11:50 AM and 2:18 PM) along I-710. The peak at 60% on the northbound side represents a localized high-density segment, as corroborated by Figure 10.

In this study, we primarily focus on the I-710 South Corridor between I-405 and State Route 47 (SR-47). Figure 10 presents four graphs, which capture both the Northbound (Left) and Southbound (Right) of I-710 South Corridor and are taken around 11:50 AM (Top) and 2:18 PM (Bottom) on June 28th, 2021 respectively. To better visualize the density plot in Figure 10, we separate the I-710 South Corridor into three major segments. Segment 1, labeled by blue box, represent I-710 freeway segment between I-405 and Willow Street. Segment 2, labeled by green box, indicates the freeway segment between SR-1 located between Willow Street and State Route 1 and Segment 3, labeled by cyan box, is located between SR-1 and Ocean Blvd. The road is segmented into about 220 100-meter segments and the number of trucks in each segment is counted and smoothed with a 500-meter Gaussian kernel where 1-sigma radius is 100 meter. According to the color scale shown in Figure 10(a) and (b), the southbound I-710 had a higher heavy-duty truck density on Segment 1 compared to the northbound. Two hours later, the southbound I-710 formed higher density values at Sections II and III, especially Section III, which is located next to the residential communities in the City of Long Beach. In Figure 11, the traffic changes from 11:50 AM to 2:18 PM is visualized. Figure 10 and 11, show highly similar traffic pattern, for example, on the northern bond direction, both snapshots have the same traffic peak in the Segment 2 and on the southern bond direction, both snapshots have a low volume part in Segment 1. Quantitatively, two snapshots correlate with *R* = 0.598*, p* < 10^−21^ at south bond direction, *R* = 1.00*, p* < 10^−30^ at north bond direction if considering the traffic peak in Segment 2, and *R* = 0.502*, p* < 10^−14^ at north bond direction if ignoring high volume areas (> 100 trucks per mile per lane). These results show both similarities and differences of truck volumes between two time of day at a high spatial resolution.

## VI. Discussion

This section first highlights the major contributions of the study. Second, the limitations of both the detection framework and the data source were discussed. Finally, this section is concluded with future research directions.

To the best of our knowledge, this study is the first to propose a hybrid framework to fuse an ensemble of detection and segmentation model with GIS information from OSM data, which effectively improve the model performance from the state-of-the-art algorithms with similar resolutions, to obtain heavy-duty trucks and shipping container distribution both on-road and off-road. This study fills the highway freight data gaps on a larger region level. The data acquired from our designed detection framework can be used to answers many important research questions. For example, from traffic data collection perspective, traditional infrastructure-based truck traffic data collection methods such as weigh-in-motions [12], piezoelectric sensors [9], and inductive loop detections [10], [11] is capable of providing a large volume of truck data with high temporal coverage. However, it is hard for those infrastructure-based traffic sensing technologies to obtain heavy-duty truck distribution in a larger region such as a long continuous truck corridor, surface streets, an off-road freight facility, and local communities. The truck detection framework developed in this study utilized the wide spatial coverage of satellite images to detect and classify heavy-duty truck located at both major freeways and surface streets, local communities, and freight facilities. The ensembled detection model is further enhanced with the GIS information extracted from OSM to refine the geographical locations of heavy-duty trucks. Thus, the proposed framework could help researchers and practitioners to better identify truck bottlenecks along continuous roadway network especially surface streets, which are lack in research due to the unavailability of detailed data. From the public health perspective, this study provides a method for estimating the distribution of trucks in communities, providing fundamental data for further examining the environmental impact of trucks (e.g. air pollution and noise) on residential areas, schools, and other sensitive land-use. Previous research mainly focused on the impact of on-road trucks on environmental health; however, given the expanding of warehouses into the residential neighborhood, it is important to examine how the off-road truck activities, including the trucks and containers in and adjacent to the warehouse properties. Further, with data from the Long Beach port area, this study shows that disadvantaged communities have three times more truck or containers per square mile comparing with the non-disadvantaged area. Also, this study finds substantial heterogeneity in truck distribution both on-road and off-road, which means that it is important for future studies to capture such heterogeneity at a high granular level to better inform transportation and public health practices.

Methodology-wise, the proposed detection framework utilized GIS information from OSM to improve the model performance on classes such as on-road heavy-duty trucks, which have weaker performance due to motion blur caused by fast-moving vehicles. The model performance on on-road heavy-duty trucks has been significantly improved through ensembling object detection model with instance segmentation model. Compared to the state-of-the-art satellite image-based truck detection algorithm, such as the one presented in [46], our framework not only detects essential vehicle locations (e.g., truck corridors, freight facilities, and local communities) but also classifies them into five crucial heavy-duty truck types. This classification capability helps fill data gaps and supports the analysis of environmental and congestion impacts of heavy-duty trucks in large areas. Moreover, our framework outperforms state-of-the-art algorithms like [44] and [46]. In terms of data, this study establishes a satellite image-based vehicle object detection dataset with an emphasis on heavy-duty trucks and the containers they carry.This dataset is the first providing truck category information with limited image resolutions. Our annotated truck data is useful for future research to further refine the truck detection or segmentation process on limited resolution satellite imagery, and also applicable to analysis of trucks in urban areas.

Despite aforementioned strength of this study, there are a few limitations. First, due to the resolution of the acquired satellite images and the motion blur issue, we were not able to identify detailed heavy-duty truck types on road. Therefore, we balanced the information needs and data quality. Second, the satellite images are only available during daytime, and at most a few times in a day. Thus, it is hard to obtain the truck density on the night time as well as continuously at a high temporal resolution, which is an inevitable limitation of this data source. Third, the study currently only focused on the area around the major seaports of Southern California. Therefore, the ground truth dataset established in this area may not be sufficiently comprehensive to understand heavy-duty trucks that are rarely occurred around the port area. In the furture, the ground truth dataset will be further enriched to incorporate various truck types and scenarios and to train a model with a better generality.

The detection framework will be further enhanced with the improvement in the image quality of the satellite image dataset. A higher resolution image can improve the localization and classification of trucks or containers, and images with fewer motion blurs can enable the classification of moving on-road trucks. Additionally, with the development of more advanced learnable ensemble methods, it is possible to further improve the ensemble performance and the model efficiency by choosing results from detection model and segmentation model based on image features instead of confidence level of each model. Finally, the proposed framework can potentially fuse with high quality infrastructure-based traffic sensing technologies such as advanced inductive loop and weigh-in-motion data (usually with temporal resolution but low spatial resolution and primarily on road only) to estimate a continuous, dense, more accurate flow of truck traffic, thus to expand the heavy-duty truck monitoring data on both spatial and temporal domains by utilizing spatiotemporal convolutional and recurrent neural networks.

## VII. Conclusion

This study developed a novel truck detection framework through the fusion of a satellite image-based object detection algorithm and a GIS-based object filter to understand port-side heavy-duty truck and shipping container distribution both on- and off-road in larger areas. First, we established a heavy-duty truck/containers-focused object dataset through the manual visual verification of every truck object on satellite images with a resolution of 0.5 meters. Second, the CenterNet model was fine-tuned and trained to identify port-side truck types and their corresponding locations. Subsequently, an ensemble of CenterNet and Mask-RCNN was used to further improve the model performance in identifying on-road heavy-duty trucks. Finally, a GIS-based object filter has been designed and applied to improve the precision of the detection. This paper also demonstrated two case studies of the proposed detection framework, which aim to understand the social justice as well as environmental impact of off- and on-road trucks respectively.

## Figures and Tables

**Fig. 12.**
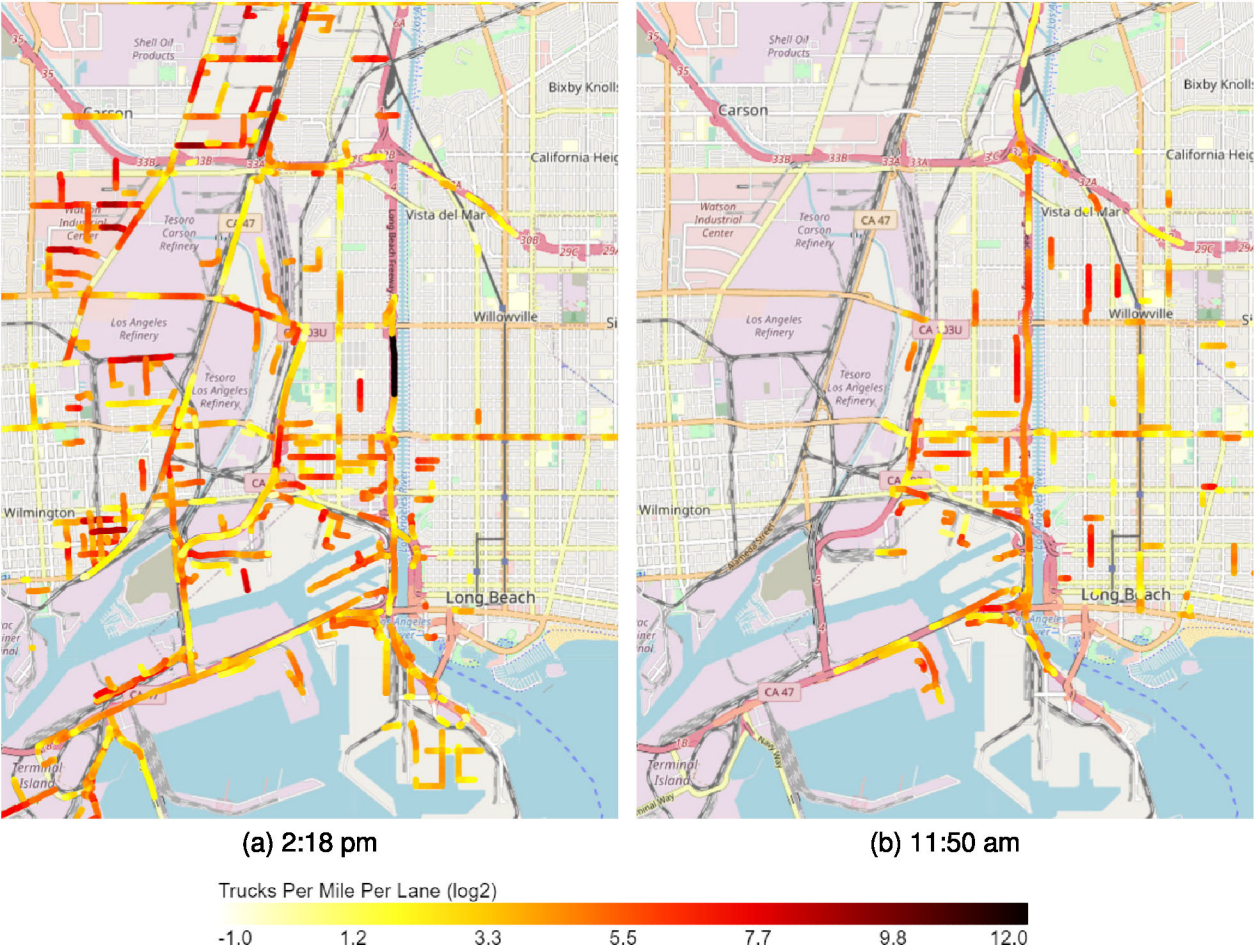
Truck distribution on road.

**TABLE I:** Satellite Image Information

	Acquired Local Time	Area	Satellite-ID	Resolution	Full Image Size
1	2021/6/28 11:50:07 PDT	Long Beach, CA	SSCI	0.5 meter	5.9km × 17.1km
2	2021/6/28 14:18:54 PDT	Long Beach, CA	SSC10	0.5 meter	7.5km × 19.4km
3	2021/9/19 14:39:38 PDT	Long Beach, CA	SSC8	0.5 meter	7.3km × 19.2km

**TABLE II:** Six Major Types of Trucks

Object Name	Description	Number in Training Samples	Number in Testing Samples
20ft Container truck (P)	20ft container tractor-trailer truck in parking lots	182	32
40–53ft Container truck (P)	40ft/45ft/53ft container tractor-trailer truck in parking lots	1,110	82
20ft Container (P)	20ft container in parking lots (standalone containers without tractor)	6,778	266
40–53ft Container (P)	40ft/45ft/53ft container in parking lots (standalone containers without tractor)	45,811	2,151
On-road Heavy-duty truck (R)	Any large tractor-trailer trucks in the roadway area (freeways, highways, local streets ...)	1,308	74
Other heavy-duty truck (P)	Other tractor-trailer trucks in parking lots	67	36
Total	-	55,356	2,641

**TABLE III:** Performance Comparison Between Different Model Variations. All Numbers Are Based on Precision and Recall Percentages. +o Means With OpenStreetMap Post-Processing and −o Means Without It. All the Evaluations Are Based on the Center Offset Standard. A Combined Row Is the Performance of the Model Without Classification. Det: CenterNet-Based Detection Model. Seg: Mask R-CNN-Based Segmentation Model. Fuse: Ensemble Performance

Model Variation	Det, −o	Det, +o	Seg, −o	Seg, +o	Fuse, −o	Fuse, +o
20ft box truck (P)	**26.5**	**26.5**	14.1	14.1	26.5	26.5
40–53ft box truck (P)	**60.8**	60.3	45.1	45.1	56.7	56.1
20ft box (P)	65.8	65.2	55.9	55.4	**65.9**	65.5
40–53ft box (P)	86.6	86.3	77.9	77.8	**88.5**	88.3
Other truck (P)	**4.53**	**4.53**	0.01	0.01	**4.53**	**4.53**
On-road truck (R)	39.1	46.2	54.5	59.9	56.4	**60.9**
Combined	86.6		79.5		**88.4**	

**TABLE IV:** Comparison of Model Performance (AP Scores) on Different Training and Validation Sets to Assess Robustness Against Domain Shifts. Random 100 Is the Reported Result in Previous Section. Image 1 Is the Result of using Entire Image 1 as Validation Set. Random 10% Is the Result of Randomly Re-Sample 10% of Tiles as Validation Set

	Random 100	Image 1	Random 10%
20ft box truck (P)	26.5	2.16	23.9
40–53ft box truck (P)	56.1	30.0	45.7
20ft box (P)	65.5	38.8	65.6
40–53ft box (P)	88.3	74.1	91.0
Other truck (P)	4.53	2.62	6.71
On-road truck (R)	60.9	51.4	61.8
Combined	88.4	75.2	89.7

**TABLE V:** Comparison of AP Scores Between Our Model and Contemporary Detection Models. Models Are Oriented R-CNN, Oriented RepPoints, and Rotated RepPoints Respectively

	Ours	ORCNN	ORP	RRP
20ft box truck (P)	**26.5**	25.0	12.2	0.3
40–53ft box truck (P)	56.1	**60.0**	54.2	5.2
20ft box (P)	**65.5**	59.2	59.5	44.3
40–53ft box (P)	**88.3**	84.0	87.9	73.2
Other truck (P)	4.5	**4.8**	2.32	0.0
On-road truck (R)	60.9	**64.2**	46.7	23.9
Combined	**88.4**	87.1	88.1	70.3

**TABLE VI:** Number of Detected Objects Per Class in the Survey Area

	Image 1	Image 3
20ft box truck (P)	8	1
40–53ft box truck (P)	116	2
20ft box (P)	1437	138
40–53ft box (P)	9353	2514
On-road truck (R)	288	68
Total number of trucks	412	71
Total number of containers	10790	2652

**TABLE VII:** Correlations Between CalEnviroScreen 4.0 Metrics and Truck/Containers Density in Image 1 Without Census Tracts With 0 Trucks (Only Metrics With More Than 0.2 or Less Than −0.2 r value). Full Table Is in Figure IX

Metric	R value	p value
Pollution Burden score	0.573	1.7048*E* – 04
Hazardous waste	0.526	6.9710*E* – 04
Underground water tank leaks	0.480	2.2719*E* – 03
Pollutants in water bodies	0.460	3.6948*E* – 03
Unemployment rate	0.426	9.5663*E* – 03
Solid waste	0.425	7.8290*E* – 03
Cleanup sites	0.399	1.3082*E* – 02
Toxic chemical levels	0.377	1.9474*E* – 02
Diesel particulate matter	0.368	2.3156*E* – 02
Poverty Population %	0.312	5.9903*E* – 02
General traffic density	0.243	1.4126*E* – 01
Population Characteristics Score	0.230	1.9021*E* – 01

**TABLE VIII:** The Comparison Between Different Detection Frameworks. All Data Are Based on the Original Work. All Frame Per Second (FPS) Values Are Linearly Scaled to the Hardware Baseline Used in Single Shot Detector (SSD) [32] to Represent the Inverse of the Computational Cost of the Model. For Inference Speed and Performance Columns, P Marks the Best-Performing Variation Reported in the Original Paper, and S Marks the Best-Speed Variation Reported in the Original Paper. All Performances Are Evaluated Using the Procedure Defined in the PASCAL Dataset [43]

Model Type	Model Name	Speed (FPS)		Performance	
		P	S	P	S
Two-Stage	R-CNN [29]	<0.1		58.5	
	Fast R-CNN [50]	0.7		70.0	
	Faster R-CNN [30]	5	7	76.4	73.2
Single-Stage	YOLO [31]	21	155	66.4	52.7
	SSD [32]	22	59	76.8	74.3
	YOLOv2 [51]	40	91	78.6	69.0
	CenterNet [24]	33	142	80.7	72.6

**Fig. 13.**
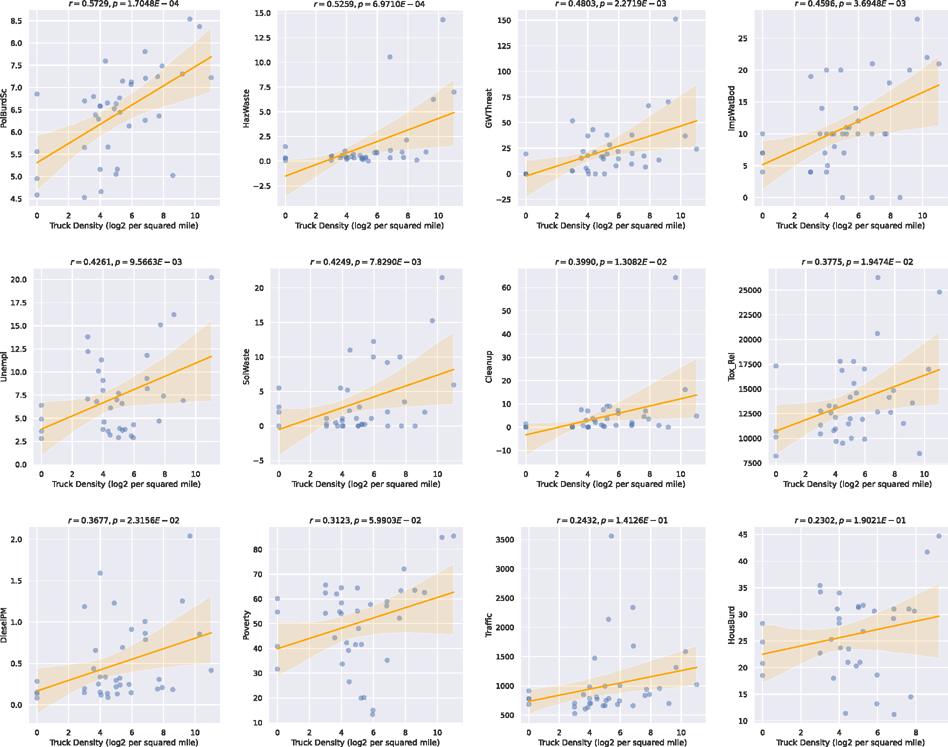
Correlation analysis over truck density against various CalEnvironScreen 4.0 metrics in Image 1. Yellow band represents 95% confidence band. Only metrics with ≥ 0.2 r-value are shown.

## Footnotes

1 Around 2,000 for R-CNN models.

## APPENDIX A MODEL BACKBONE SELECTION

In Table VIII, we compared multiple baseline detection models for performance and inference speed on PASCAL VOC [43] dataset. All the inference speed numbers are linearly scaled according to the performance comparison in their original paper.

## APPENDIX C ON-ROAD TRUCK TRAFFIC ANALYSIS

In Table IX, the correlations between log_2_ of truck/container density including 0 density areas and the selected 23 CalEnviroScreen 4.0 metrics are shown. 12 of their fitted linear models are shown in Figure 13.

**TABLE IX:** Correlations Between CalEnviroScreen 4.0 Metrics and Truck/Containers Density

Metric	R value	p value
Pollution Burden score	0.573	1.7048*E* – 04
Hazardous waste	0.526	6.9710*E* – 04
Underground water tank leaks	0.480	2.2719*E* – 03
Pollutants in water bodies	0.460	3.6948*E* – 03
Unemployment rate	0.426	9.5663*E* – 03
Solid waste	0.425	7.8290*E* – 03
Cleanup sites	0.399	1.3082*E* – 02
Toxic chemical levels	0.377	1.9474*E* – 02
Diesel particulate matter	0.368	2.3156*E* – 02
Poverty Population %	0.312	5.9903*E* – 02
Pop. Characteristics Score	0.280	9.8674*E* – 02
General traffic density	0.243	1.4126*E* – 01
House burden	0.230	1.9021*E* – 01
Low birth weight rate	0.197	2.4958*E* – 01
Heart attacks rate	0.172	3.0057*E* – 01
Limited English speaking	0.164	3.3903*E* – 01
Undereducated population	0.143	4.0502*E* – 01
PM2.5	0.115	4.9289*E* – 01
Ozone concentration	−0.100	5.5176*E* – 01
Asthma rate	0.068	6.8714*E* – 01
Risk of lead exposure	−0.029	8.6865*E* – 01
Pesticide ingredients (lbs)	0.010	9.5106*E* – 01
Drinking water contaminant	0.000	1.0000*E* + 00

In Figure 12, density of on-road trucks are shown for every road with truck detected.
